# Lessons Learned from a Military–Biotechnology Partnership to Develop a Broad-Spectrum Small-Molecule Inhibitor for Snakebite Envenoming

**DOI:** 10.3390/toxins18040180

**Published:** 2026-04-08

**Authors:** Kendra L. Lawrence, Jeffery L. Owen, Lindsey S. Garver, Brandi A. Ritter, Christopher M. Wilson, Ginger R. Boatright, F. Y. Bowling, Timothy F. Platts-Mills, Andrea K. Renner, Rebecca W. Carter

**Affiliations:** 1 Warfighter Protection & Acute Care, Operational Medical Systems, Defense Health Agency, Fort Detrick, MD 21702, USA; 2 Ophirex, Inc., 5643 Paradise Drive, Suite 2, Corte Madera, CA 94925, USA; 3 U.S. Special Operations Command, Tampa, FL 33621, USA

**Keywords:** snake bite, varespladib, phospholipase A2, translation of innovation

## Abstract

Snakebite envenoming causes an estimated 138,000 deaths annually worldwide, with approximately 75% of fatalities occurring prior to arrival at definitive medical care. Even in regions where antivenom is available in hospitals, the absence of treatment options before a victim can reach definitive care results in delays of many hours before therapy is initiated. Manufacturing complexity, region-specific products, and the risk of anaphylaxis further limit the availability and use of antivenom in many regions. Reducing the persistently high mortality of snakebite envenoming requires both novel scientific approaches and partnerships that extend beyond traditional disciplinary and funding silos. This article describes the collaboration between Ophirex, a Public Benefit Corporation developing the oral secretory phospholipase A2 (sPLA2) inhibitor varespladib, and the United States military, which has identified a capability gap in snakebite treatment for forward-deployed personnel. The partnership was driven by a shared requirement for a shelf-stable, easy-to-administer, snake-species-agnostic therapy suitable for use prior to definitive medical care. A central insight of the program was that military operational requirements and global public health needs converged around the same product characteristics, enabling a strategically aligned development effort. From early proof-of-concept studies through regulatory pathway definition and advanced development, the Military–Ophirex partnership integrated operational requirements, regulatory planning, and iterative risk mitigation to advance manufacturing, nonclinical, and clinical development. This work provides both practical insights into complex drug development and a case study in how structured partnerships can carry innovation through translation in underfunded and operationally challenging conditions.

## 1. Historical Precedents for Military–Pharmaceutical Industry Partnerships

Changing the treatment paradigm for any disease requires scientific innovation, sustained capital, and regulatory navigation. The challenges are even greater for diseases that are time-sensitive and biologically complex. In these settings, military–pharmaceutical partnerships have historically accelerated progress by identifying operational gaps, coordinating resources, and enabling translation when civilian systems alone were insufficient. The development of penicillin during World War II is a well-described example of such a partnership. While Alexander Fleming discovered penicillin and Florey and Chain advanced its purification, large-scale production required coordinated U.S. military funding, industrial standardization, and rapid fermentation scale-up through pharmaceutical partners. The military did not replace industry; it de-risked and accelerated it [1,2].

Because deployed service members encounter pathogens, flora, and fauna that many Americans do not, the U.S. military has a long history of investing in development of counter measures against tropical diseases and leveraging industry partnerships. For example, decades of such collaboration in research and manufacture of antimalarial treatments and prophylaxis contributed to nearly a dozen licensed drugs used to fight malaria on both military and humanitarian fronts, most recently tafenoquine, approved in 2018 [3].

Similarly, pyridostigmine bromide extended release was approved in 2024 for the pre-exposure treatment of soman nerve agent poisoning following a development partnership between Amneal Pharmaceuticals, Inc. (Bridgewater, NJ, USA) and the Department of Defense Chemical and Biological Defense Program [4]. Sagramostim was approved in 2018 for the treatment of myelosuppression following radiation exposure as a result of a development partnership between Partner Therapeutics, Inc. (Lexington, MA, USA) and the Biomedical Advanced Research and Development Authority, a branch of the U.S. Department of Health and Human Services responsible for development of medical countermeasures [5].

The recurring principle in these partnerships is that innovation may originate in academia or industry, but translation into a development program that can obtain FDA approval often requires strategic partnership and aligned investment from a government entity.

## 2. Setting the Context: Snakebite Envenoming

Snakebite envenoming causes approximately 138,000 deaths annually and leaves hundreds of thousands more with permanent disability. Although advances in antivenom and hospital-based care have improved outcomes for patients who reach treatment, an estimated 75% of fatalities occur before hospital arrival [6], underscoring the limited ability of intravenously administered therapies to reduce overall mortality and the persistent unmet need in the pre-hospital setting [7,8,9,10,11].

Snakebite presents unusual biological and operational complexity. Approximately 300 medically important venomous snake species exist globally, with both inter- and intra-species variation in venom composition [12]. Envenoming results in a diverse range of toxicities including life-threatening neurotoxicity and coagulopathy as well as disabling soft-tissue injuries [13]. Operational realities for both military and civilian populations further complicate care. Snakebites occur unpredictably, often in rural or austere environments lacking rapid access to advanced care.

Early intervention is critically important to optimize outcomes for snakebite, and multiple studies identify time from bite to care as a key predictor of severity, with delays associated with worse outcomes [7,8,14], but the only existing pharmacologic therapy, antivenom, must be given intravenously and its use is limited primarily to hospitals. Antivenom has additional limitations including species or regional specificity, high manufacturing complexity and cost, risk of anaphylaxis, and requires knowledgeable medical staff for appropriate administration [13,15,16].

Snakebite therapeutics have historically been underfunded. Grants typically support discovery or early-stage research rather than the capital-intensive path to regulatory approval and scalable manufacturing. In part this is because snakebite envenoming falls between the cracks. It is not an infectious disease, so it has not been recognized as a priority by major funders such as the Gates Foundation or listed under the FDA’s Tropical Disease Priority Review Voucher. The scale of the global public health burden, comparable to or exceeding several other neglected tropical diseases, has been underappreciated. Additionally, the scientific and regulatory complexity of achieving approval for a paradigm-shifting therapy in such a heterogeneous, toxin-mediated condition further discourages investment.

Despite these challenges, unlike many neglected tropical diseases, snakebite affects people in higher-income countries, including the United States, Australia, and parts of Europe and Asia, where hospitals create well-defined commercial markets. A time-of-bite therapy prescribed in anticipation of a snakebite expands this market beyond hospital-based treatment alone. Thus, although snakebite is neglected, there is a meaningful commercial opportunity for new treatments. For early- to mid-stage development companies, the involvement of a committed development partner may enable progression towards this commercially sustainable market.

## 3. Ophirex’s Approach: Varespladib

Ophirex, Inc. (Corte Madera, CA, USA) is pursuing a small-molecule strategy targeting secretory phospholipase A_2_ (sPLA2), a toxin family present in the venoms of many medically significant snakes and implicated in systemic toxicity. Varespladib, an orally bioavailable sPLA2 inhibitor, was selected as the lead candidate [17,18]. Its intended role is not to replace antivenom but to serve as an early intervention administered at or near the time-of-bite to reduce systemic toxicity and delay progression until definitive care can be delivered. Venom sPLA2s are present in more than 95% of the world’s venomous snake species, making sPLA2 an attractive broadly conserved target for a species-agnostic therapeutic approach [12,19].

Varespladib was originally developed by Shionogi & Co., Ltd. (Osaka, Japan) and Eli Lilly and Company (Indianapolis, IN, USA) as an inhibitor of sPLA2. It is a substituted indole small molecule, and its oral prodrug, varespladib methyl, was developed to enable oral administration. Prior non-snakebite development included 29 clinical studies in more than 4600 subjects across indications including sepsis and acute coronary syndrome, providing substantial prior human clinical experience before repurposing for snakebite [20].

Later studies, including delayed-rescue and large-animal models, as well as combination studies with antivenom or other toxin inhibitors, further strengthened the translational rationale [21,22,23,24].

The mechanistic rationale was pragmatic: inhibition of a broadly conserved toxin family offers the potential for cross-species activity without requiring snake identification. Structural, in vitro, and in vivo studies support this approach, showing that varespladib binds conserved hydrophobic-channel and calcium-binding regions of venom sPLA2s [25,26,27] and can inhibit lethality and morbidity across diverse snake species [14,15,16,17,18]. A robust body of basic science research over the past four decades has elucidated the contribution of sPLA2 to a broad range of toxicities and, in particular, its contributions to neuromuscular weakness and vascular collapse following snakebite [28,29]. The preclinical evidence has progressed from broad venom in vitro screening to delayed-rescue studies, large-animal models, and studies in combination with antivenom or other toxin inhibitors, further strengthening the translational rationale [24,30,31,32,33].

Recognizing the potential of varespladib to address a global public health problem, Ophirex was formed as a Public Benefit Corporation with a charter that establishes a commitment to making treatment affordable and accessible in low- and middle-income countries. The solution identified by Ophirex was of interest to the U.S. military because varespladib is orally bioavailable, has a well-defined safety profile based on earlier clinical research for other indications, and has the potential to offer sufficient breadth of efficacy to yield survival benefits for snake species in key geographic regions.

## 4. U.S. Military Interest: Different Use Case, Same Required Characteristics

For U.S. warfighters, primarily troops on the ground, antivenom remains the standard of care for snakebite envenoming but presents significant logistical and operational challenges. Most antivenoms are species- or region-specific, which complicates the procurement process and many lack FDA approval, further complicating the process and timeliness of procurement. Additionally, most antivenoms require cold-chain storage or reconstitution from lyophilized formulations and must be administered intravenously by trained personnel capable of managing potential hypersensitivity reactions, including anaphylaxis. The number of vials required for effective treatment is often unpredictable, and for many snake species, antivenom availability is limited or nonexistent.

A central insight of the partnership was that the operational requirements of the military and the needs of global public health were closely aligned. Both contexts require a therapy that can be administered orally or through a simple route, used at or near the time-of-bite, remain stable in extreme climates, and have broad-spectrum activity without the need for snake identification.

To address this need, the Defense Health Agency (DHA) conducted market research to assess existing therapeutic approaches and identify companies capable of advancing a candidate through regulatory approval. Prospective solutions were evaluated against criteria aligned with warfighter requirements to demonstrate sufficient scientific maturity and a credible pathway to final product availability. Commercial viability was explicitly identified as a key criterion for both the technology and the development partner. The anticipated military market alone would be insufficient to sustain high-quality, long-term manufacturing capacity, and reliance solely on military procurement, which may be subject to variability based on operational demands would not provide a stable foundation for commercialization.

This alignment directly shaped development priorities. Although there was an intravenous formulation of varespladib, which would likely be of value to patients in whom neurotoxicity has progressed to the point that they are unable to swallow a pill, development efforts have focused on the oral formulation which will be an appropriate early treatment for almost all patients. Further, although varespladib has demonstrated benefit for specific snake venoms even when given after the onset of severe neurotoxicity [31,34], development focused on early intervention and breadth of toxin inhibition across multiple species. The overlap in military and global health requirements reduced strategic tension: a therapy optimized for special operations use would also address morbidity and mortality in rural and resource-limited civilian environments.

## 5. Evolution of the Military–Ophirex Partnership

In 2012, the U.S. Air Force Special Operations Command identified a capability gap in the treatment of snakebite envenoming in deployed settings. This gap was recognized by U.S. Special Operations Command (USSOCOM) and in 2017 by the DHA Small Business Innovation Research (SBIR) program. Exploratory support began through USSOCOM and Air Force channels, then advanced through feasibility and proof of concept then continued research and development through the SBIR program. Early stages were high risk and focused on feasibility and mechanism validation. As predefined development criteria were met, the program transitioned into advanced development within the Broad-Spectrum Snakebite Antidote (BSSA) framework, where regulatory alignment and technology readiness became central priorities.

Once the development reached a technology readiness level suggesting that regulatory approval was possible, DHA Operational Medical Systems (OPMED) recognized the capability gap within the Joint Forces. All deployed personnel would benefit from a shelf-stable, lightweight, easy-to-administer, venomous snake species-agnostic therapy suitable for austere environments. A particular need was identified for special operations forces, which frequently operate in remote settings with limited evacuation capability and prolonged timelines to reach advanced medical care. In this context, DHA leadership recognized the importance of an early, field-administered treatment capable of reducing mortality and long-term morbidity prior to definitive care.

In 2019, the Broad-Spectrum Snakebite Antidote (BSSA) Program was initiated to address this capability gap. The program’s objective is to support development of an FDA-approvable therapy suitable for far-forward use, reducing the logistical and administrative burdens associated with antivenom while enabling earlier intervention following envenoming and decreasing the risk of mortality and morbidity.

With support from DHA OPMED and the SBIR program, Ophirex advanced manufacturing, formulation, and nonclinical development activities in parallel, enabling progression of varespladib into Phase 2 clinical evaluation. This period also allowed military and Ophirex leadership to establish a shared understanding of operational requirements and regulatory constraints, align on program objectives, and build the disciplined communication and mutual trust necessary to execute a complex dual-use development program.

This foundation of shared objectives, operational clarity, and disciplined communication proved essential as clinical development encountered operational challenges inherent to snakebite envenoming. Unlike many drug development programs that target relatively uniform disease mechanisms within conventional care settings, snakebite envenoming is biologically heterogeneous, with severity influenced by venom composition and dose. Further, sPLA2, the target toxin, acts rapidly. Accordingly, evaluation of varespladib as an early, time-of-bite treatment required a development strategy fundamentally different from conventional hospital-based adjunctive therapy studies conducted hours after envenoming and following antivenom administration.

It became clear during development that conventional hospital-based clinical studies would be inherently limited in their ability to support approval of a therapy intended for administration at or near the time-of-bite, before patients reach definitive care. In this context, a regulatory strategy integrating human safety and clinical data with animal efficacy data offered a more appropriate evidentiary framework. Interpreting these findings and translating them into a viable regulatory path required sustained, tightly integrated coordination between military and Ophirex leadership, with iterative review of alternatives, risk, evidentiary sufficiency, and mitigation strategies to ensure alignment with Ophirex and military requirements.

Advancing a first-in-class, field-administered therapy for a heterogeneous toxin-mediated condition demanded agreement not only on scientific direction but also on the evidentiary standard required for approval. As a result of the coordinated strategic review with the military, Ophirex engaged the U.S. Food and Drug Administration (FDA) regarding the Animal Rule pathway, designed for conditions in which human efficacy trials are not ethical or feasible. At the same time, the military added varespladib to the Medical Product Priority List, via Defense Healthy Agency Medical Product Acceleration Committee. Codified under Public Law 115-92, it increases Department of War (DoW) collaboration with FDA to meet the urgent medical material needs of the warfighter and facilitates communication between the DoW and FDA on the Sponsor’s behalf.

The FDA agreed that the Animal Rule pathway is appropriate for a time-of-exposure intervention for which human efficacy trials are impractical. However, using the Animal Rule does not eliminate or reduce evidentiary rigor. It requires well-characterized and representative animal models, robust demonstration of efficacy in those models, with studies held to the standard of pivotal human clinical trials, and adequate human safety data. Thus, while this pathway provides a viable route forward, it remains scientifically demanding and resource intensive, with integrated development across manufacturing, nonclinical, clinical, and regulatory domains.

The collaborative framework established early in the program remains central to ongoing execution, enabling the partners to manage scientific, regulatory, and operational complexity as development advances toward addressing a critical unmet need in both global public health and military operational readiness.

## 6. Lessons Learned

The collaboration has yielded several practical lessons that may be applicable to other mission-driven therapeutic programs.

1. Early and transparent communication are crucial

Scientific and operational challenges are part of drug development and must be surfaced early to enable joint problem-solving.

2. Commercial viability is important

With limited exceptions, military procurement alone cannot sustain pharmaceutical manufacturing. A viable civilian market supports the viability of the company and assures military access to the drug once approved.

3. Operational needs can inform development

Clear articulation and iterative refinement of U.S. military operational requirements sharpen development priorities.

4. Regulatory flexibility is essential

Traditional trial paradigms in which all patients receive a hospital-based standard of care treatment may not capture meaningful benefit in neglected or operationally complex pre-hospital or austere conditions.

5. Shared mission reduces friction

When military and global health objectives align, the partnership becomes durable.

6. Contractual clarity protects partnership integrity

Clear agreements regarding deliverables, intellectual property, and fiduciary responsibility preserve optionality and reduce the risk that unforeseen challenges undermine the partnership.

## 7. Conclusions and Path Forward

Snakebite envenoming is a complex problem to address because it sits at the intersection of biological heterogeneity, time sensitivity, geographic dispersion, and historic underinvestment. The military–Ophirex partnership emerged not simply from shared interest but from structural alignment: operational military requirements and global public health needs converged around the same desired product characteristics. That alignment, combined with regulatory strategic planning and attention to commercial viability, has enabled a viable development pathway. Although regulatory approval has not yet been achieved, the remaining steps are now clearly defined under the Animal Rule framework. If varespladib is approved, military collaboration will extend beyond regulatory authorization to facilitate adoption across operational units, including stakeholder engagement, user training, and incorporation into Clinical Practice Guidelines.

Realizing the full potential of varespladib will require additional strategic partnerships. Ensuring availability in rural regions of low- and middle-income countries will depend on collaboration with in-country regulators, clinicians, health systems, procurement bodies, and public health programs. Further expansion of therapeutic breadth, potentially through combination with inhibitors targeting other major venom toxin classes, could broaden and strengthen the value proposition of an oral, time-of-bite intervention. Advancing this next phase of development will require coordinated scientific, development, and implementation partnerships.

Snakebite illustrates a broader principle: neglected diseases are not inherently undevelopable. When mission-driven demand, commercial sustainability, and regulatory strategy are deliberately aligned, progress becomes achievable. Durable solutions require more than innovation alone; they require structured partnerships capable of carrying that innovation through translation.

## Data Availability

The original contributions presented in this study are included in the article. Further inquiries can be directed to the corresponding author(s).
